# Sequence variants with large effects on cardiac electrophysiology and disease

**DOI:** 10.1038/s41467-019-12682-9

**Published:** 2019-10-22

**Authors:** Kristjan Norland, Gardar Sveinbjornsson, Rosa B. Thorolfsdottir, Olafur B. Davidsson, Vinicius Tragante, Sridharan Rajamani, Anna Helgadottir, Solveig Gretarsdottir, Jessica van Setten, Folkert W. Asselbergs, Jon Th. Sverrisson, Sigurdur S. Stephensen, Gylfi Oskarsson, Emil L. Sigurdsson, Karl Andersen, Ragnar Danielsen, Gudmundur Thorgeirsson, Unnur Thorsteinsdottir, David O. Arnar, Patrick Sulem, Hilma Holm, Daniel F. Gudbjartsson, Kari Stefansson

**Affiliations:** 1 deCODE genetics/Amgen Inc., Reykjavik, Iceland; 2 School of Engineering and Natural Sciences, University of Iceland, Reykjavik, Iceland; 3 Department of Cardiology, Division Heart & Lungs, University Medical Center Utrecht, Utrecht University, Utrecht, The Netherlands; 4 Institute of Cardiovascular Science, Faculty of Population Health Sciences, University College London, London, UK; 5 Health Data Research UK and Institute of Health Informatics, University College London, London, UK; 6 Department of Internal Medicine, Akureyri Hospital, Eyrarlandsvegur, 600 Akureyri, Iceland; 7 Department of Paediatric Cardiology, Children’s Hospital, Landspitali-The National University Hospital of Iceland, Reykjavik, Iceland; 8 Department of Family Medicine, University of Iceland, Reykjavik, Iceland; 9 Department of Development, Primary Health Care of the Capital Area, Reykjavik, Iceland; 10 Division of Cardiology, Internal Medicine Services, Landspitali-The National University Hospital of Iceland, Reykjavik, Iceland; 11 Faculty of Medicine, School of Health Sciences, University of Iceland, Reykjavik, Iceland

**Keywords:** Genome-wide association studies, Quantitative trait, Cardiovascular genetics, Cardiology

## Abstract

Features of the QRS complex of the electrocardiogram, reflecting ventricular depolarisation, associate with various physiologic functions and several pathologic conditions. We test 32.5 million variants for association with ten measures of the QRS complex in 12 leads, using 405,732 electrocardiograms from 81,192 Icelanders. We identify 190 associations at 130 loci, the majority of which have not been reported before, including associations with 21 rare or low-frequency coding variants. Assessment of genes expressed in the heart yields an additional 13 rare QRS coding variants at 12 loci. We find 51 unreported associations between the QRS variants and echocardiographic traits and cardiovascular diseases, including atrial fibrillation, complete AV block, heart failure and supraventricular tachycardia. We demonstrate the advantage of in-depth analysis of the QRS complex in conjunction with other cardiovascular phenotypes to enhance our understanding of the genetic basis of myocardial mass, cardiac conduction and disease.

## Abstract (web summary)

Aberrant morphology of the QRS complex in an electrocardiogram can be associated with cardiac morbidity and mortality. Here, the authors perform genome-wide association studies for ten measures of the QRS complex in 81,192 individuals and find 86 previously unreported loci that associate with at least one parameter.

## Introduction

**Fig. 1:**
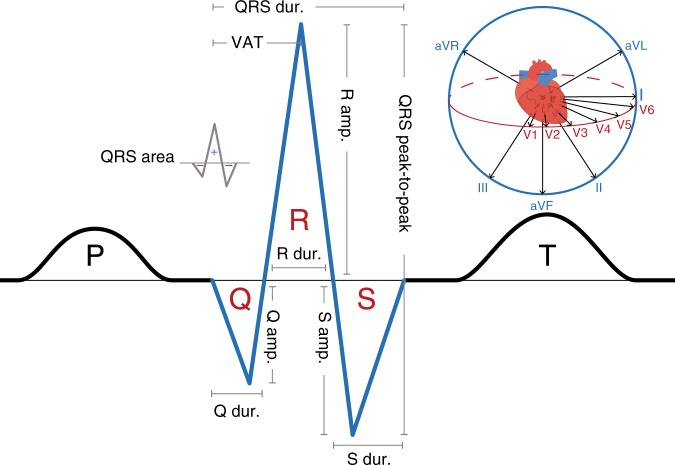
ECG wave and the QRS complex. We denote QRS measures used in the analysis with grey lines. We illustrate the different viewpoints of the 12 ECG leads in the upper-right corner. Amp. amplitude, Dur. duration, VAT ventricular activation time

The QRS complex (Fig. 1) of the electrocardiogram (ECG) is a summation of electrical activity in the heart during ventricular depolarisation. It represents electrical activation of the left and right ventricles of the heart, propagated through the specialised conduction system^1^, which includes the His bundle, right and left bundle branches and the Purkinje network. Normal ventricular depolarisation is a rapid process activating different ventricular myocardial segments in a precise temporal sequence, resulting in a narrow QRS complex with a characteristic pattern on the 12-lead ECG^1^. Deviation from the norm has been associated with sudden cardiac arrest (prolonged ventricular activation time)^2,3^ and mortality in the general population (prolonged QRS duration)^4^ and among individuals free of cardiovascular disease (low QRS voltage)^5^.

Many traits and diseases affect the morphology of the QRS complex, including body habitus, primary conduction abnormalities, hypertrophy and dilatation of the ventricles, myocardial infarction, pericardial effusion and lung disease^6^. Measures of the QRS complex have been used as prognostic indicators and markers of heart disease severity, such as heart failure^7^, ventricular hypertrophy^8^ and amyloidosis^9^.

Prior genome-wide association studies (GWAS) of the QRS complex were limited to testing QRS duration and voltage criteria reflecting left ventricular hypertrophy, yielding sequence variants at 58 loci with minor allele frequency (MAF) >4%^10–15^.

To gain a better understanding of the molecular mechanism of ventricular conduction, we perform a large GWAS of ten measures of the QRS complex in 12 leads from ECGs of 81,192 individuals. These ten measures are the Q, R and S wave amplitudes and durations, QRS complex area and duration, ventricular activation time, and the distance from the peak of the R wave to the nadir of the S wave (QRS p-2-p). We analyse each measure for 12 ECG leads: limb leads (I–III), augmented limb leads (aVR, aVL, aVF) and precordial leads (V1–V6). We also analyse three other parameters: mean QRS duration over 12 leads, the Cornell voltage criterion and the Sokolow-Lyon voltage criterion. In total, we analyse 123 QRS complex parameters and this results in the identification of 190 associations between the QRS complex and variants at 130 loci, for which we further assess effects on seven echocardiographic traits and 22 cardiovascular diseases. We demonstrate the advantage of analysing 12 leads of the ECG and identify rare protein-coding variants in genes that can improve the understanding of risk and progression of heart disease and help direct future studies.

## Results

### QRS associations

We performed a GWAS on 81,192 individuals, testing 32.5 million common and rare (MAF > 0.01%) SNPs and indels for association with 123 QRS complex parameters (Supplementary Fig. 1, Supplementary Tables 1 and 2). The variants were identified by whole-genome sequencing 15,220 Icelanders and imputed into 151,677 long-range phased individuals and their relatives^16^. We adjusted the threshold for genome-wide significance with a weighted Bonferroni procedure, using as weights the predicted functional impact of association signals^17^ (Supplementary Table 3). Sequence variants at 130 loci (MAF > 0.1%) associate with at least one QRS complex parameter. Using conditional analysis, we identified 60 secondary signals at 32 of the loci, resulting in 190 distinct associations (Supplementary Data 1 and 2). We replicated associations at all 54 reported QRS loci with at least one of the 123 QRS parameters (Supplementary Data 3, Supplementary Table 4; *P* < 0.05/123 = 4.1 × 10^−4^).

Among the 190 variants that associate with QRS parameters, 159 are common (MAF > 5%) with effects ranging in magnitude from 0.04 to 0.14 standard deviations (s.d.), 14 are low-frequency (1% < MAF ≤ 5%) with effects ranging from 0.11 to 0.35 s.d., and 17 are rare (MAF ≤ 1%) with effects ranging from 0.19 to 1.9 s.d.

We found genome-wide significant associations with 115 of 123 QRS parameters tested. There were no genome-wide significant associations with eight Q wave amplitude and duration parameters, of which four had relatively few available measurements (*N* < 15,000). Of the 190 variants, 160 associate genome-wide significantly with more than one QRS parameter. The R amplitude is the QRS measure that captures the most associations (*N* = 96), followed by QRS area (*N* = 85) and R duration (*N* = 76), while the Cornell voltage criterion captures the fewest (*N* = 18) (Supplementary Fig. 2). Some of the variants associate only with the R amplitude (*N* = 16), R duration (*N* = 9), S amplitude (*N* = 9), QRS area (*N* = 5), QRS p-2-p (*N* = 4) or ventricular activation time (*N* = 4).

**Fig. 2:**
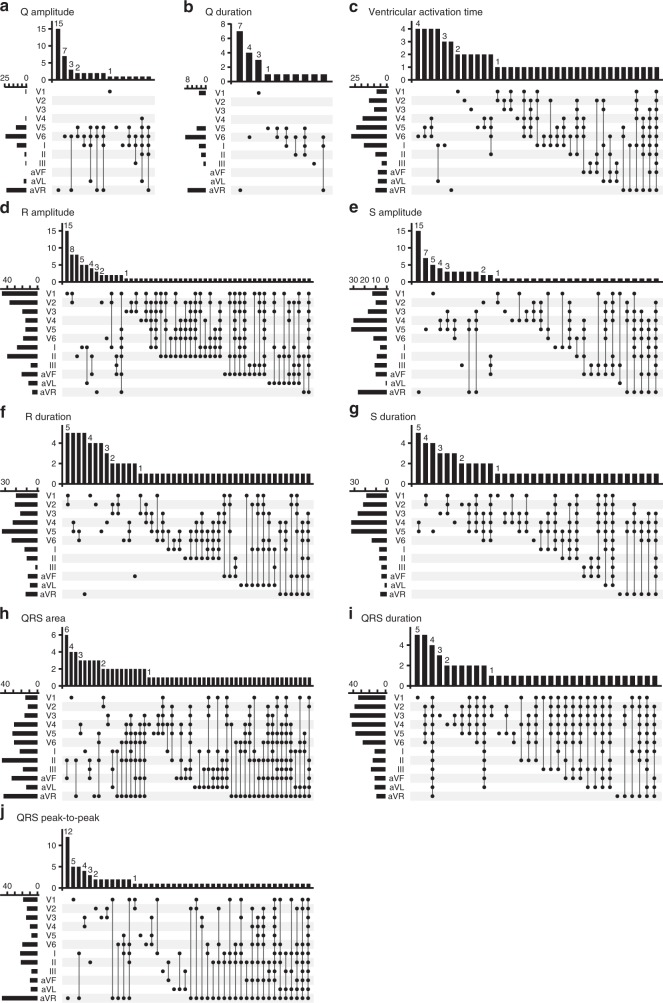
Intersection plots of associations for the QRS measures. In each panel, the left bar plot shows how many of the 190 QRS variants associate (*P* < 5 × 10^−8^) with each lead. The top bar plot shows the sizes of intersecting sets of associations for the leads denoted with (connected) black dots. **a** Q amplitude; **b** Q duration; **c** Ventricular activation time; **d** R amplitude; **e** S amplitude; **f** R duration; **g** S duration; **h** QRS area; **i** QRS duration; **j** QRS peak-to-peak

For the R amplitude, we identified most associations with lead V1 (*N* = 47, Fig. 2). Fifteen variants associate only with the R amplitude in lead V1. We identified more than twice as many associations for the QRS p-2-p amplitude in lead aVR than in any other lead (*N* = 47). For the QRS area, we identified most associations with lead II (*N* = 43); with lead V3 for QRS duration (*N* = 39); with lead V5 for R duration (*N* = 33), S duration (*N* = 33) and S amplitude (*N* = 31); and with lead V6 for ventricular activation time (*N* = 28), Q amplitude (*N* = 24) and Q duration (*N* = 9). Across all QRS measures, we observed the fewest associations with leads III and aVL.

The genes harbouring the missense and loss-of-function variant associations likely encode proteins that play roles in the biology of the QRS complex. Variants annotated as coding/splice represent 47 of the 190 QRS associations, of which 28 are at unreported QRS loci—14 rare and 7 low frequency (Supplementary Data 1 and 2). Loss-of-function variants in *HMCN2* and *SH3BGR* associate most significantly with the QRS p-2-p amplitude, in *MYBPC3* and *TTN* with R wave duration and *SCN5A* with QRS duration. *MYBPC3*, *TTN* and *SCN5A* are established cardiac disease genes, but *SH3BGR* and *HMCN2* are not. Of the missense variants, 20 associate most significantly with the R amplitude (in *ADAMTS7*, *ALPK3*, *BAG3*, *CASP7*, *CCDC141*, *CILP*, *DERL3*, *FHOD3*, *FLNC*, *MYH6*, *MYH7*, *MYH7B*, *MYO18B*, *NACA*, *PLEC*, *RYR2*, *STAB1* and *TTN*), 4 with the QRS area (in *ALDH1A2*, *RBM20* and *TTN*) and 4 with the QRS p-2-p amplitude (in *CAND2*, *HMCN2*, *SENP2* and *STON1-GTF2A1L*). Twelve variants associate most significantly with other QRS parameters (in *ADAMTS6*, *ADPRHL1*, *C17orf58*, *CCDC141*, *CFAP46*, *KLHL38*, *LAMA3*, *MAPT*, *PLCE1*, *SCN10A*, *SYNPO2L* and *TTN*).

We tested the 190 QRS variants for association with the ventricular conduction disorders, left anterior/posterior fascicular block (LAFB/LPFB) and left/right bundle branch block (LBBB/RBBB). Using a 5% false discovery rate (FDR, *P* ≤ 0.003), we observed 46 associations with 37 variants (Supplementary Data 4). Three variants associate genome-wide significantly with LAFB, including the missense variant p.Leu294Arg (MAF = 4.4%) in *ADPRHL1* (OR = 1.37, *P* = 4.8 × 10^−8^), coding for a cardiac-restricted protein essential for heart chamber outgrowth^18^. The stop-gain variant p.Ser699Ter in *HMCN2* (MAF = 2.0%) associates with a high risk of LPFB (OR = 2.31, *P* = 5.2 × 10^−4^).

To assess the genetic relationship between ventricular depolarisation and other electrical functions of the cardiac conduction system, we tested the genetic correlation between different ECG measures. The genetic correlation (Methods) between mean QRS duration and mean PR interval duration is 0.14 (95% CI: 0.06–0.21) and between mean QRS duration and mean QT interval duration is 0.31 (95% CI: 0.23-0.40). We also tested the 190 QRS variants for association with ECG measures reflecting atrial and atrioventricular conduction (P–Q component of the ECG), and ventricular repolarisation (ST–T component) (Supplementary Fig. 3, Supplementary Data 5). Using a 5% FDR (*P* ≤ 0.016), 177 of the variants associate with a non-QRS measure of the ECG. Of those, 16 have more significant effects on non-QRS components: the PR interval (at *CAV1*, *OBFC1*, *SCN5A*, *SCN10A* and *TBX3*), T amplitude (at *KLF12*, *LMF1*, *MYBPC3* and *RNF207*), P amplitude (at *SYNPO2L* and *MYH6*) and ST duration (at *MYH7B* and *RNF207*). The pattern of magnitude and direction of effects associated with different ECG parameters is highly variable between variants.

### Replication

We assessed the associations of unreported QRS variants in (1) QRS parameters similar to ours from 19,885 ECGs of participants in the UK Biobank and (2) a published GWAS^14^ on four QRS measures from up to 73,518 participants.

Using data from the published GWAS on QRS duration and three voltage criteria^14^, we tested variants that associated genome-wide significantly in the Icelandic material with any of these four parameters. We tested seven variants and all replicated (*P* < 0.05 and the same direction of effects, Supplementary Data 6). In the smaller UK Biobank data, we tested variants that we expected to replicate (over 99% power). Of 13 variants tested, all had the same direction of effects in both datasets and 11 replicated (*P* < 0.05 and the same direction of effects, Supplementary Data 7).

### Associations with echocardiographic traits

Using echocardiograms of 21,275 Icelanders, we tested the QRS variants for association with 7 echocardiographic left ventricular measurements (Supplementary Table 5, Supplementary Data 8). We observed 23 associations with 18 variants (Table 1, 5% FDR, *P* ≤ 7.1 × 10^−4^), most with the left ventricular end-diastolic diameter (LVEDD). The splice-acceptor site mutation c.927-2 A > G in *MYBPC3*, known to cause familial hypertrophic cardiomyopathy^19^, associates with interventricular septum thickness (*β* = 1.2 s.d., *P* = 1.5 × 10^−37^), left ventricular posterior wall thickness and LVEDD. The missense variant p.Cys151Arg in *BAG3*, known to associate with heart failure due to dilated cardiomyopathy^20^, associates with LVEDD (*β* = −0.10 s.d., *P* = 1.8 × 10^−13^). We discovered ten unreported associations, including an association with rs4794562[T] intronic to *HLF*, encoding a circadian PAR bZip transcription factor^21^, and LVEDD (*β* = −0.06 s.d., *P* = 5.3 × 10^−6^).

**Table 1:** Associations between the QRS variants and echocardiographic traits

Trait	Variant	Chr.	Pos. (hg38)	EA/OA	EAF (%)	Locus	Annotation	Top QRS parameter	Effect (s.d.)	*P*
ARD	rs11874	17	46,939,827	A/G	12.9	*GOSR2*	3′ UTR	S dur., V4	0.11	8.0 × 10^−9^
ARD	rs7543039	1	99,584,092	C/T	48.8	*PALMD*	Intergenic	S amp., V4	−0.064	1.5 × 10^−8^
ARD	rs2687938	13	50,180,110	T/C	45.6	*DLEU1*	Intergenic	QRS area, V4	0.057	4.6 × 10^−7^
ARD	rs335196	5	123,184,837	A/G	43	*PRDM6*	Intronic	QRS area, I	−0.055	1.8 × 10^−6^
ARD	rs34766086*	2	40,512,632	T/G	5.67	*SLC8A1*	Upstream	R dur., V5	−0.085	7.1 × 10^−4^
EF	rs2234962	10	119,670,121	C/T	24.2	*BAG3*	Missense	R amp., V1	0.051	1.7 × 10^−4^
EF	rs72692220*	1	50,974,791	A/G	3.15	*CDKN2C*	Downstream	Mean QRS dur.	−0.13	1.8 × 10^−4^
EF	rs35006907	8	124,847,575	A/C	35.8	*ZNF572*	Intergenic	R amp., V1	0.042	6.0 × 10^−4^
LVEDD	rs2234962	10	119,670,121	C/T	24.2	*BAG3*	Missense	R amp., V1	−0.099	1.8 × 10^−13^
LVEDD	rs397516082	11	47,346,372	C/T	0.175	*MYBPC3*	Splice acceptor	R dur., V4	−0.57	2.3 × 10^−9^
LVEDD	rs35006907	8	124,847,575	A/C	35.8	*ZNF572*	Intergenic	R amp., V1	−0.058	9.7 × 10^−7^
LVEDD	rs4794562*	17	55,302,450	T/C	25.7	*HLF*	Intronic	QRS dur., V3	−0.06	5.3 × 10^−6^
LVEDD	rs17305149*	3	14,235,797	G/A	18.5	*LSM3*	Intergenic	R dur., V5	0.064	1.8 × 10^−5^
LVEDD	rs72692220*	1	50,974,791	A/G	3.15	*CDKN2C*	Downstream	Mean QRS dur.	0.14	4.0 × 10^−5^
LVEDD	rs16866538	2	178,795,185	A/G	4.93	*TTN*	Missense	S amp., V1	−0.098	2.3 × 10^−4^
LVEDD	rs143158900*	10	112,746,619	CA/C	17.6	*VTI1A*	Intronic	QRS p-2-p, I	0.055	2.6 × 10^−4^
LVEDD	rs147436240	6	36,680,287	C/CGCGT	17.2	*CDKN1A*	Intronic	Mean QRS dur.	−0.055	3.9 × 10^−4^
LVEDD	rs9909004*	17	66,310,015	C/T	38.6	*PRKCA*	Intronic	QRS dur., V3	0.041	5.5 × 10^−4^
LVESD	rs1461990*	8	107,075,400	G/C	47.5	*ANGPT1*	Intergenic	Q amp., V6	0.07	5.8 × 10^−4^
LVPWT	rs397516082	11	47,346,372	C/T	0.175	*MYBPC3*	Splice acceptor	R dur., V4	0.62	4.9 × 10^−11^
IVST	rs397516082	11	47,346,372	C/T	0.175	*MYBPC3*	Splice acceptor	R dur., V4	1.2	1.5 × 10^−37^
IVST	rs4284441*	12	114,924,167	T/C	36.5	*TBX3*	Intergenic	QRS area, V6	0.047	7.2 × 10^−5^
IVST	rs7301677*	12	114,943,342	T/C	34.8	*TBX3*	Intergenic	VAT, V6	−0.041	5.7 × 10^−4^

We only show results for 5% FDR, *P* ≤ 7.1 × 10^−4^. We denote unreported associations with a star. *EA* effect allele, *OA* other allele, *EAF* effect allele frequency, *s.d.* standard deviation, *Dur.* duration, *Amp.* amplitude, *ARD* aortic root diameter (*N* = 19,513), *EF* ejection fraction (*N* = 17,109), *LVEDD* left ventricular end-diastolic dimension (*N* = 18,487), *LVESD* left ventricular end-systolic dimension (*N* = 5704), *LVPWT* left ventricular posterior wall thickness (*N* = 18,626), *IVST* interventricular septum thickness (*N* = 18,775)

### Associations with cardiovascular diseases

The 190 QRS variants are a priori more likely to associate with cardiovascular diseases than random sequence variants, and thus we tested them for association with 22 cardiovascular diseases in Icelandic datasets, including cardiac arrhythmias, cardiomyopathy and coronary artery disease (CAD). For 18 of the diseases, we also had data from the UK Biobank^22^ and performed a meta-analysis (Supplementary Table 6, Supplementary Data 9).

Two rare QRS variants associate with several cardiovascular dysfunctions. C.927-2A>G in *MYBPC3* (MAF = 0.18%) associates with cardiomyopathy, heart failure, ventricular tachycardia and atrial fibrillation (AF), and p.Arg721Trp (MAF = 0.34%) in *MYH6* with sick sinus syndrome (SSS), AF and coarctation of the aorta. We have previously reported the effects of these variants on risk and progression of heart diseases, as well as two other QRS variants at *PLEC* and *PALMD*^19,23–26^. Excluding these four variants, we observed 91 associations with 57 of the remaining QRS variants (5% FDR, *P* ≤ 1.0 × 10^−3^). Most of the associations are with AF (*N* = 30), essential hypertension (*N* = 11), CAD (*N* = 10) and myocardial infarction (*N* = 10), of which many have been reported^27–34^. We also identified reported associations with dilated cardiomyopathy and heart failure^20^, SSS and presence of pacemaker^10^.

We found 41 unreported associations with 32 variants and 16 cardiovascular traits (Table 2). Notably, the intronic variant rs11166990[A] in *PXN*, which associates with the R amplitude in lead V1, associates with reduced risk of AF (MAF = 1.3%, OR = 0.82, *P* = 4.0 × 10^−8^). Paxillin, encoded by *PXN*, is expressed at focal adhesions of non-striated cells and costameres of striated muscle cells^35^. Rs11166990[A] upstream of *PTK2* (coding for focal adhesion kinase), which also associates with the R amplitude in lead V1, associates with hypertension (MAF = 48%, OR = 0.97, *P* = 1.1 × 10^−6^), aortic valve stenosis (OR = 0.92, *P* = 2.1 × 10^−4^), and is known to associate with reduced risk of AF. The rare missense variant p.Ala2397Val in *FLNC* (MAF = 0.13%), which associates with a larger R amplitude in lead V1, reduces the risk of myocardial infarction (OR = 0.42, *P* = 1.3 × 10^−4^) and delays its onset (*β* = 0.78 s.d., *P* = 1.1 × 10^−4^), with greater protection against myocardial infarction in individuals younger than 76 years (OR = 0.21, *P* = 3.8 × 10^−6^). Variants in *FLNC* are known to cause cardiomyopathy, and we recently reported the frameshift variant p.Phe1626Serfs*40 in *FLNC*^36^ with opposite direction of effects on the R amplitude in lead V1 and myocardial infarction to those of p.Ala2397Val (Supplementary Table 7).

**Table 2:** Unreported associations between the QRS variants and cardiovascular diseases

Trait	Chr.	Pos. (hg38)	EA/OA	Locus	Comb. OR (95% CI)	Comb. *P*	*P*_het_	Ice OR	Ice *P*	Ice EAF (%)	UKB OR	UKB *P*	UKB EAF (%)
AVS	8	140,653,873	A/G	*PTK2*	0.92 (0.87, 0.96)	2.1 × 10^−4^	0.29	0.89	7.0 × 10^−4^	48	0.94	0.066	49.2
AF	12	120,230,731	A/G	*PXN*	0.82 (0.76, 0.88)	4.0 × 10^−8^	0.86	0.83	0.01	1.28	0.81	1.2 × 10^−6^	2.25
AF	6	22,570,808	G/A	*HDGFL1*	1.04 (1.02, 1.07)	1.2 × 10^−4^	0.97	1.04	0.023	23.1	1.04	0.002	27.3
AF	6	117,168,041	T/G	*VGLL2*	1.04 (1.02, 1.06)	1.8 × 10^−4^	0.15	1.02	0.29	38.6	1.05	1.1 × 10^−4^	38.9
AF	17	67,991,933	T/C	C17orf58*	1.04 (1.02, 1.06)	2.2 × 10^−4^	0.45	1.05	0.0046	24.9	1.03	0.013	27.8
AF	2	238,142,909	T/C	*KLHL30*	0.95 (0.92, 0.98)	5.7 × 10^−4^	0.66	0.95	0.082	9.89	0.94	0.0027	11.2
AF	5	154,492,281	G/C	*HAND1*	1.03 (1.01, 1.05)	7.9 × 10^−4^	0.46	1.02	0.15	35.9	1.04	0.0018	36.7
CAVB	2	178,905,448	A/C	CCDC141*	1.29 (1.17, 1.42)	3.6 × 10^−7^	0.56	1.26	3.3 × 10^−4^	15.3	1.33	2.6 × 10^−4^	13.8
CAVB	17	55,302,450	T/C	*HLF*	0.86 (0.79, 0.94)	7.8 × 10^−4^	0.14	0.91	0.094	25.7	0.8	0.0011	26.7
CAVB	6	118,400,465	A/G	*CEP85L*	1.13 (1.05, 1.22)	8.1 × 10^−4^	0.49	1.11	0.031	45.8	1.17	0.0079	55.3
CMCS	10	33,233,881	G/A	*NRP1*	1.30 (1.14, 1.50)	1.7 × 10^−4^	0.66	1.27	0.016	6.29	1.35	0.0035	7.8
CMCS	2	54,665,663	G/AS	*SPTBN1*	0.84 (0.77, 0.92)	2.0 × 10^−4^	0.45	0.87	0.023	26.5	0.81	0.0024	27.1
CAD	12	114,924,167	T/C	*TBX3*	1.03 (1.02, 1.05)	2.8 × 10^−5^	0.27	1.04	6.5 × 10^−4^	36.5	1.03	0.0078	39
CAD	1	170,662,622	T/C	*PRRX1*	1.03 (1.01, 1.04)	6.9 × 10^−4^	0.8	1.03	0.026	47.9	1.02	0.01	45.7
DCM	3	12,807,323	T/C	CAND2*	0.82 (0.74, 0.90)	3.9 × 10^−5^	0.87	0.81	0.013	32.7	0.82	0.001	33.7
DCM	8	124,847,575	A/C	*ZNF572*	0.82 (0.74, 0.90)	7.0 × 10^−5^	0.75	0.84	0.035	35.8	0.81	7.2 × 10^−4^	31
DCM	11	47,253,513	A/G	*NR1H3*	1.19 (1.08, 1.31)	3.1 × 10^−4^	0.99	1.19	0.03	31.8	1.19	0.0041	27.4
HF	17	55,302,450	T/C	*HLF*	0.95 (0.92, 0.97)	6.7 × 10^−5^	0.57	0.95	0.005	25.7	0.94	0.0039	26.7
HF	4	113,508,251	T/C	*CAMK2D*	1.04 (1.02, 1.07)	0.001	0.45	1.04	0.028	27	1.06	0.01	24.7
HT	8	140,653,873	A/G	*PTK2*	0.97 (0.96, 0.98)	1.1 × 10^−6^	0.47	0.97	0.0028	48	0.98	9.4 × 10^−5^	49.2
HT	12	56,720,316	A/G	NACA*	1.02 (1.01, 1.04)	1.7 × 10^−4^	0.95	1.02	0.075	22.7	1.02	9.4 × 10^−4^	27.3
HT	11	47,253,513	A/G	*NR1H3*	0.98 (0.97, 0.99)	2.7 × 10^−4^	0.47	0.99	0.25	31.8	0.98	4.1 × 10^−4^	27.4
HT	2	174,603,041	T/C	*WIPF1*	1.02 (1.01, 1.04)	4.6 × 10^−4^	0.12	1	0.78	22.3	1.03	1.3 × 10^−4^	20.6
HT	8	9,152,396	A/G	*PPP1R3B*	1.02 (1.01, 1.03)	6.3 × 10^−4^	0.89	1.02	0.13	26.8	1.02	0.0021	31.3
HT	8	11,932,112	T/G	*DEFB136*	0.96 (0.94, 0.98)	6.4 × 10^−4^	1	0.96	6.4 × 10^−4^	32.5	—	—	—
HCM	8	124,847,575	A/C	*ZNF572*	1.31 (1.14, 1.52)	2.1 × 10^−4^	0.23	1.24	0.018	35.8	1.5	0.002	31
IS	11	19,986,122	T/A	*NAV2*	1.07 (1.03, 1.11)	7.9 × 10^−4^	0.7	1.06	0.021	24.5	1.08	0.014	23.4
MI	7	128,856,555	T/C	FLNC*	0.42 (0.27, 0.66)	1.3 × 10^−4^	1	0.42	1.3 × 10^−4^	0.134	—	—	—
MI	2	36,454,050	A/G	*CRIM1*	1.03 (1.01, 1.05)	4.1 × 10^−4^	0.28	1.02	0.12	37.4	1.04	8.0 × 10^−4^	40.8
MI	18	36,652,647	T/C	FHOD3*	0.97 (0.95, 0.99)	0.001	0.84	0.96	0.019	30.1	0.97	0.021	28.6
PMI	2	178,905,448	A/C	CCDC141*	1.12 (1.06, 1.18)	5.9 × 10^−5^	0.91	1.12	0.0018	15.3	1.11	0.011	13.8
PMI	1	6,212,076	G/GA	*RNF207*	0.91 (0.86, 0.96)	6.2 × 10^−4^	1	0.91	6.2 × 10^−4^	36.5	—	—	—
PMI	4	113,508,251	T/C	*CAMK2D*	1.08 (1.03, 1.12)	8.7 × 10^−4^	0.08	1.11	2.5 × 10^−4^	27	1.03	0.38	24.7
PDA	5	65,470,971	A/G	ADAMTS6*	4.88 (1.90, 12.55)	0.001	1	4.88	0.001	0.245	—	—	—
PMC	12	114,943,342	T/C	*TBX3*	0.84 (0.75, 0.93)	9.7 × 10^−4^	1	0.84	9.7 × 10^−4^	34.8	—	—	—
SSS	4	113,508,251	T/C	*CAMK2D*	1.11 (1.05, 1.18)	1.2 × 10^−4^	0.76	1.11	4.7 × 10^−4^	27	1.14	0.1	24.7
SSS	2	178,905,448	A/C	CCDC141*	1.13 (1.06, 1.21)	2.1 × 10^−4^	0.22	1.15	9.6 × 10^−5^	15.3	1.01	0.89	13.8
SVT	1	111,987,808	G/A	*KCND3*	1.61 (1.38, 1.88)	2.7 × 10^−9^	0.79	1.57	1.7 × 10^−4^	2.21	1.64	3.7 × 10^−6^	1.38
SVT	1	170,662,622	T/C	*PRRX1*	1.11 (1.06, 1.17)	8.6 × 10^−6^	0.76	1.12	0.0047	47.9	1.11	5.6 × 10^−4^	45.7
SVT	1	115,749,108	C/T	*CASQ2*	0.92 (0.88, 0.96)	4.4 × 10^−4^	0.36	0.89	0.0052	43.1	0.93	0.02	44.6
SVT	2	178,856,319	A/G	CCDC141*	1.14 (1.06, 1.23)	7.9 × 10^−4^	0.36	1.09	0.18	11.7	1.17	0.0013	8.53

We show results for 5% FDR, *P* ≤ 0.001. We denote coding variants with a star. *EA* effect allele, *OA* other allele; *P*_*het*_ *P* value for heterogeneity in the effect estimates between the Icelandic and UK Biobank data, *EAF* effect allele frequency, *AVS* aortic valve stenosis, *AF* atrial fibrillation, *CAVB* complete atrioventricular block, *CMCS* congenital malformations of cardiac septa, *CAD* coronary artery disease, *DCM* dilated cardiomyopathy, *HF* heart failure, *HT* hypertension, *HCM* hypertrophic cardiomyopathy, *IS* ischaemic stroke, *MI* myocardial infarction, *PMI* pacemaker insertion, *PDA* patent ductus arteriosus, *PMC* perimyocarditis, *SSS* sick sinus syndrome, *SVT* supraventricular tachycardia

We found four unreported associations with supraventricular tachycardia (SVT), represented by rs75013985[G] intronic to *KCND3* (AF = 2.2%, OR = 1.61, *P* = 2.7 × 10^−9^); rs629234[T] upstream of *PRRX1* (MAF = 47.9%, OR = 1.11, *P* = 8.6 × 10^−6^), which also associates with AF and CAD; rs12144451[C] in *CASQ2* (MAF = 43.1%, OR = 0.92, *P* = 4.4 × 10^−4^), which also associates with AF; and the missense variant p.Arg935Trp in *CCDC141* (MAF = 11.7%, OR = 1.14, *P* = 7.9 × 10^−4^). We found three associations with complete AV block (CAVB), represented by p.Glu382Asp in *CCDC141* (MAF = 15.3%, OR = 1.29, *P* = 3.6 × 10^−7^), which also associates with pacemaker insertion (OR = 1.12, *P* = 5.9 × 10^−5^) and SSS (OR = 1.13, *P* = 2.1 × 10^−4^); rs4794562[T] intronic to *HLF* (MAF = 25.7%, OR = 0.86, *P* = 7.8 × 10^−4^), which also associates with heart failure (OR = 0.95, *P* = 6.7 × 10^−5^); and rs17226667[A] near *CEP85L* (MAF = 45.8%, OR = 1.13, *P* = 8.1 × 10^−4^). We found three unreported associations with dilated cardiomyopathy, represented by the missense variant p.Val77Ala in *CAND2* (MAF = 32.7%, OR = 0.82, *P* = 3.9 × 10^−5^), rs35006907[A] near *ZNF572* (MAF = 35.8%, OR = 0.82, *P* = 7.0 × 10^−5^), and rs10838681[A] upstream of *NR1H3* (MAF = 31.8%, OR = 1.19, *P* = 3.1 × 10^−4^).

Some of the QRS variants that associate with AF have effects on non-QRS components of the ECG, mainly the P amplitude and the PR interval, measuring atrial and atrioventricular nodal conduction (Supplementary Fig. 3). We tested these QRS variants in a subset of our data containing 236,896 ECGs in sinus rhythm of 57,399 individuals without AF. Most variants still had the strongest effects on the QRS complex and non-significant effects on the P amplitude (5% FDR), indicating that the QRS effect is not a consequence of the arrhythmia (Supplementary Fig. 4).

### Parent-of-origin effects

We tested the 190 QRS variants for a difference in effects under maternal and paternal models of transmission (Supplementary Table 8)^37^. Two variants have more than three times greater effect when inherited paternally than maternally (Table 3, *P* < 0.05/190 = 2.6 × 10^−4^). One is rs11761424[A] (MAF = 35%) intronic to *DGKB* (encoding a diacylglycerol kinase), which associates most significantly with R wave duration in lead V4 (*β*_pat_ = 0.068 s.d., *β*_mat_ = 0.018 s.d., *P*_het_ = 4.1 × 10^−5^). Sequence variants near *DGKB* are known to associate with type 2 diabetes^38^ and AF^27^ but do not correlate with rs11761424 (*r*^2^ < 0.01). The other is rs116904997[A] (MAF = 1.3%) intronic to *PXN* (encoding paxillin), which associates most significantly with the R amplitude in lead V1 (*β*_pat_ = 0.28 s.d., *β*_mat_ = 0.073 s.d., *P*_het_ = 1.6 × 10^−4^). Paxillin is a cytoskeletal protein involved in actin-membrane attachment at sites of focal adhesion^35^. In the Genotype-Tissue Expression (GTEx) dataset^39^, rs116904997[A] associates more significantly than any other variant with the expression of *PXN* in the left ventricle of the heart (Supplementary Fig. 5, *P* = 3.0 × 10^−9^). These loci are not known to be imprinted.

**Table 3:** QRS variants with parent-of-origin effects

Top QRS parameter	Variant	Chr.	Pos. (hg38)	EA/OA	EAF (%)	Locus	Pat. effect (s.d.)	Pat. *P*	Mat. effect (s.d.)	Mat. *P*	*P*_het_
R dur., V4	rs11761424	7	14,414,730	A/G	35	*DGKB*	0.068	6.5 × 10^−15^	0.018	0.034	4.1 × 10^−5^
R amp., V1	rs116904997	12	120,230,731	A/G	1.28	*PXN*	0.28	7.3 × 10^−13^	0.073	0.062	1.6 × 10^−4^

We show results for *P*_het_ < 0.05/190 = 2.6 × 10^−4^. *EA* effect allele, *OA* other allele, *EAF* effect allele frequency, *Pat*. paternal, *s.d.* standard deviation, *Mat.* maternal, *P*_*het*_ *P* value for heterogeneity in the effect estimates between the paternal and maternal models, *Dur*. duration, *Amp*. amplitude

### Pathway analysis

We applied the data-driven expression-prioritised integration for complex traits (DEPICT, Methods)^40^ to search for tissues and cell types in which our variants are more likely to be expressed. Enrichment testing of expression in 209 tissues and cell types pointed out cardiovascular tissue (atria, ventricles and arteries) as well as the myometrium and muscles (1% FDR, Supplementary Data 10). Furthermore, DEPICT identified 495 enriched gene sets out of 14,461 reconstituted gene sets (1% FDR, Supplementary Data 11). After clustering gene sets by similarity, the most significant gene sets related to abnormal myocardium layer morphology, pericardial effusion, abnormal heart morphology and the cell-substrate adherens junction. Many genes near unreported QRS variants were part of gene sets linked to the adherens junction, including *VCL*, *SMTN*, *PXN*, *FHL2*, *SVIL* and *ANKRD1*. We also used DEPICT to point out genes within each associated locus based on functional similarity to genes within other associated loci (5% FDR, Supplementary Data 12).

Of the 190 QRS variants, 20 are intronic or coding in genes with higher expression in heart muscle than other tissue types in the Human Protein Atlas (total 201 genes)^41^. Variants in some of these genes have been described by OMIM as causing autosomal dominant heart conditions (*MYBPC3*, *MYH6*, *MYH7*, *CTNNA3*, *RBM20*, *RYR2*, *SCN5A*) or associated in GWAS with heart diseases (*CASQ2*, *MYH7B*, *MYO18B*, *SYNPO2L*, *TBX5*, *TRIM63*)^27,42,43^, resting heart rate (*CCDC141*, *FHOD3*)^44^ or non-QRS ECG parameters (*ALPK3*, *RNF207*)^45,46^. Other genes have not been implicated in heart diseases (*ADPRHL1*, *KLHL38*, *SH3BGR*). Given both the QRS association and the heart muscle expression, variants in these genes could substantially affect cardiac function.

Using the prior information on expression of genes in the heart, we searched for additional rare (MAF ≤ 1%) coding variants that associate with the QRS complex in these 201 genes. Thirteen additional variants associate with one or more QRS parameters (Supplementary Data 13, *P* < 0.05/(number of coding variants tested) = 2.4 × 10^−5^). We found four missense variants at unreported QRS loci: *C10orf71*, *FBXO40*, *GRM1* and *HCN4*. We note that *HCN4* is a known bradycardia gene^47^. We identified one frameshift and three missense QRS variants where we had identified non-coding QRS variants previously in our study: in *CTNNA3*, *NKX2-5*, *RNF207* and *TBX5*. We recently reported the missense variant p.Phe145Leu in *NKX2-5* as causing cardiomyopathy and arrhythmias^36^.

## Discussion

We used whole-genome sequence data to perform a GWAS on the QRS complex of the 12-lead ECG in 81,192 Icelanders and identified 130 QRS loci. We found 190 distinct QRS variants, of which 106 are at 86 loci that have not been reported for QRS before, and assessed their effects on seven echocardiographic traits and 22 cardiovascular diseases.

We demonstrate an advantage of performing an in-depth examination of the QRS complex when studying the genetic influence on ventricular depolarisation, myocardial mass and associated cardiac disease. Our approach yielded 130 QRS loci, compared to 52 loci identified in a recent GWAS of the QRS complex^14^ of a similar size (up to 73,518 individuals) that assessed only the QRS duration and three voltage criteria, measures commonly used clinically. If we had exclusively tested these same four measures, we would have identified associations at only 54 loci. Thus, additional measures of the QRS complex (the QRS area, ventricular activation time and distinct measures of the Q, R and S waves) yielded 76 additional loci.

Many of the 123 parameters tested are correlated, and most variants associate with more than one. However, some variants associate with only one QRS measure, some with only one lead. The R amplitude is the QRS measure that captures most QRS associations, about half of them. The R wave in lead V1 captures half of the R amplitude associations, and 15 variants associate only with the R amplitude in lead V1. Under normal conditions, the R wave is small in lead V1 but gets progressively larger in the precordial leads, usually reaching maximum amplitude in lead V5. The small R wave in lead V1 represents the initial part of ventricular depolarisation. Some conditions may cause an abnormally large R wave in lead V1, including right bundle branch block, type A Wolf–Parkinson–White Syndrome, posterior myocardial infarction, right ventricular hypertrophy and acute right ventricular dilatation^48^. The QRS area captures the second highest number of associations. Although the QRS area has not been used in a GWAS before, it improves identification of left ventricular hypertrophy over standard voltage criteria^49^. The genetic influence on various aspects of the cardiac conduction system is both tightly linked and complex, as the majority of QRS variants associates also with ECG measures reflecting atrial and atrioventricular conduction, and ventricular repolarisation, but with a variable magnitude and direction of effects.

Before our study, only two low-frequency (1% ≤ MAF ≤ 5%) and no rare variants had been shown to associate with the QRS complex in a GWAS^14^. We found 33 unreported large-effect associations with rare or low-frequency coding variants, including 13 associations identified by using a priori expression information. This includes associations with a missense variant in *ADPRHL1* that also associates genome-wide significantly with left anterior fascicular block, and a stop-gain variant in *HMCN2* that also associates with a high risk of left posterior fascicular block. The rare missense variant p.Ala2397Val in *FLNC* associates with a larger R amplitude in lead V1 and reduced risk of myocardial infarction, with opposite direction of effects to those of p.Phe1626Serfs*40, a reported frameshift variant that causes cardiomyopathy, thus suggesting an opposite functional effect of p.Ala2397Val^36^. *FLNC* encodes filamin C, a large actin-crosslinking protein expressed in striated muscles. Filamin C anchors major protein complexes at the sarcolemma, Z-discs and intercalated discs in cardiomyocytes to the actin cytoskeleton and provides a scaffold for a variety of cytoplasmic signalling proteins^50,51^.

We identified 91 associations for 57 of the QRS variants with a spectrum of cardiovascular diseases, including arrhythmias and conduction disorders, hypertension, ischemic disease, cardiomyopathies, and valve diseases. We found 41 unreported associations, including four with supraventricular tachycardia (SVT), one of which is with a low-frequency variant located near *KCND3*, a potassium channel gene that has been implicated in Brugada syndrome^52^ and AF^27^, and is the first genome-wide significant SVT association.

We found an association with an intronic variant in *PXN*, encoding paxillin, which has not been associated with AF before. Paxillin is expressed at focal adhesions of non-striated cells and costameres of striated muscle cells^35^, and regulates cardiac contractility in the zebrafish^53^. The *PXN* variant, as well as a variant intronic to *DGKB*, associates primarily with the QRS complex when the allele is inherited paternally. These loci are not known to be imprinted, although paxillin has been shown to regulate expression, not imprinting, of the imprinted genes *IGF2* and *H19* through long-range chromosomal interactions between the *IGF2* and *H19* promoters and a shared distal enhancer^54^.

A common variant in *HLF* associates with a decreased risk of heart failure, a lower risk of CAVB and RBBB, a smaller ventricle and a shorter QRS duration. *HLF* encodes a member of the proline acidic-rich (PAR) protein family, a subset of the bZIP transcription factors that accumulate with robust circadian rhythms in tissues with high amplitudes of clock gene expression. The knockout of *HLF* along with two other PAR bZip transcription factors has been shown to lead to cardiac hypertrophy and left ventricular dysfunction in mice^55^.

In summary, our results demonstrate the advantage of analysing the QRS complex in a detailed manner. The use of whole-genome sequencing facilitates the discovery of associations with protein-coding variants that implicate particular genes in the biology of ventricular depolarisation and cardiac pathology. The findings provide new insights into the complex genetics of cardiac electrophysiology and will help direct future functional studies of cardiovascular diseases.

## Methods

### Icelandic data

The ECGs were performed between 1998 and 2015 at Landspitali—The National University Hospital, the sole tertiary care hospital in Iceland. They were done in both inpatient and outpatient setting, using the Philips PageWriter Trim III, Philips PageWriter 200, Philips PageWriter 50 and Philips PageWriter 70 cardiographs, and stored in the Philips TraceMasterVue ECG Management System. We analysed ten measures of the QRS complex from 405,732 ECGs of 81,192 individuals. These ten measures were the Q, R and S wave amplitudes and durations; QRS complex area and duration; ventricular activation time (VAT, time between the onset of the Q wave and the peak of the R wave); and the distance from the peak of the R wave to the nadir of the S wave (QRS peak-to-peak amplitude). We analysed each measure for 12 ECG leads: limb leads (I–III), augmented limb leads (aVR, aVL, aVF) and precordial leads (V1–V6). In addition, we analysed mean QRS duration over 12 leads, the Cornell voltage criterion (for men: (R amplitude in lead aVL + S amplitude in lead V3) × QRS duration; for women: (R amplitude in lead aVL + S amplitude in lead V3 + 0.8 mV) × QRS duration) and the Sokolow-Lyon voltage criterion ((S amplitude in lead V1 + R amplitude in lead V6) × QRS duration). We also used four phenotypes derived from automated ECG diagnoses: left anterior fascicular block (LAFB), left bundle branch block (LBBB), left posterior fascicular block (LPFB) and right bundle branch block (RBBB).

We used seven phenotypes describing measurements from echocardiograms: aortic root diameter (*N* = 19,513), ejection fraction (*N* = 17,109), left ventricular end-diastolic diameter (*N* = 18,487), left ventricular end-systolic diameter (*N* = 5,704), mitral regurgitation (*N* = 5,291), left ventricular posterior wall thickness (*N* = 18,626) and interventricular septum thickness (*N* = 18,775). We obtained these measurements from a database of 53,122 echocardiograms from 27,460 individuals, read by cardiologists at LUH between 1994 and 2015.

deCODE genetics has extensive medical information on cardiovascular diseases for use in association analyses. The QRS variants were tested for association with 22 cardiovascular diseases: aortic valve stenosis (*N* = 2457), atrial fibrillation (*N* = 14,710), coarctation of the aorta (*N* = 119), complete atrioventricular block (*N* = 1008), congenital malformations of cardiac septa (*N* = 1884), coronary artery disease (*N* = 38,918), dilated cardiomyopathy (*N* = 424), heart failure (*N* = 15,237), hypertension (*N* = 44,290), hypertrophic cardiomyopathy (*N* = 372), ischemic stroke (*N* = 5626), mitral valve disease (*N* = 940), myocardial infarction (*N* = 24,691), pacemaker insertion (*N* = 3578), patent ductus arteriosus (*N* = 628), perimyocarditis (*N* = 971), pre-excitation Wolff-Parkinson-White syndrome (*N* = 275), sick sinus syndrome (*N* = 3568), sudden cardiac death (*N* = 3128), supraventricular tachycardia (*N* = 1461), tetralogy of fallot (*N* = 60) and ventricular tachycardia (*N* = 945). The sample sets were based on discharge diagnoses from LUH from 1987 to 2017. The controls consisted of disease-free controls (up to 380,000), randomly drawn from the Icelandic genealogical database, and individuals from other genetic studies at deCODE.

The Icelandic Data Protection Authority and the National Bioethics Committee of Iceland (no. VSNb2015030024/03.01 and VSNb2015030022/03.01) approved the study.

### Genotyping

In Iceland, 32.5 million sequence variants (MAF > 0.01%) were identified by whole-genome sequencing 15,220 Icelanders using Illumina standard TruSeq methodology to a mean depth of 35× (s.d. 8X) and imputed into 151,677 chip-typed individuals and their first- and second-degree relatives^16,56^. In the UK Biobank, genotyping was performed using a custom-made Affimetrix chip (UK BiLEVE Axiom)^57^ in the first 50,000 participants and Affimetrix UK Biobank Axiom array in the remaining participants^58^ (95% of the signals were on both chips). Wellcome Trust Centre for Human Genetics performed the imputation using a combination of 1000 Genomes phase 3 (ref. ^59^), UK10K^60^ and HRC^61^ reference panels, for up to 92.5 million SNPs.

### Statistical analysis

We adjusted quantitative traits for sex and age at measurement, transformed them into a standard normal distribution using a rank-based inverse normal transformation, and used a linear mixed model implemented by BOLT-LMM^62^ to test them for association with genotypes. We used a logistic regression model to test binary traits for association with genotypes. For the deCODE data, we adjusted for sex, county of birth, current age or age at death (first- and second-order terms included), blood sample availability for the individual, and an indicator function for the overlap of the lifetime of the individual with the timespan of phenotype collection. For the UK Biobank data, we used 40 principal components to adjust for population stratification and adjusted for age and sex in the logistic regression model. The UK Biobank study included only white British individuals. In each study, we used LD score regression^63^ to account for inflation in test statistics due to cryptic relatedness and stratification. To combine the deCODE and UK Biobank results, we used a fixed-effect inverse variance method based on effect estimates and standard errors^64^. We used a likelihood-ratio test to compute all *P* values. In conditional analyses, we tested variants within ±1 Mb from the top variant and with correlation *r*^2^ < 0.05 with the top variant, and required the variants to be genome-wide significant after correcting for the top variant in a regression model.

### Genetic correlation

We estimated the genetic correlation between pairs of traits using LD score regression (v.1.0.0)^65^ and pre-computed LD scores for European populations (downloaded from https://data.broadinstitute.org/alkesgroup/LDSCORE/eur_w_ld_chr.tar.bz2).

### Depict

We used DEPICT^40^ to (1) prioritise candidate causal genes at associated loci, (2) highlight enriched pathways, and (3) identify tissues/cell types where genes at associated loci are highly expressed. DEPICT uses gene expression data derived from a panel of 77,840 mRNA expression arrays together with 14,461 existing gene sets based on molecular pathways derived from experimentally verified protein–protein interactions^66^, genotype–phenotype relationships from the Mouse Genetics Initiative^67^, Reactome pathways^68^, KEGG pathways^69^, and Gene Ontology (GO) terms^70^. DEPICT reconstitutes these 14,461 gene sets by calculating for each gene the probability of membership in each gene set, based on similarities across the expression data. Using these membership probabilities and a set of trait-associated loci, DEPICT tests whether any of the 14,461 reconstituted gene sets are enriched for genes at the trait-associated loci, and prioritises genes that share predicted functions with genes at other trait-associated loci. Additionally, DEPICT uses 37,427 human mRNA microarrays to search for tissues/cell types in which genes from associated loci are highly expressed (all genes harbouring variants in LD of *r*^2^ > 0.5 from the most significant variant). We ran DEPICT using all QRS-associated variants. We also used DEPICT to compute pairwise Pearson correlations between all reconstituted gene sets and clustered them by similarity using the Affinity Propagation method^71^.

### Reporting summary

Further information on research design is available in the Nature Research Reporting Summary linked to this article.

## Supplementary information

Reporting Summary

Supplementary Information

Peer Review File

Description of Additional Supplementary Files

Supplementary Data 1

Supplementary Data 2

Supplementary Data 3

Supplementary Data 4

Supplementary Data 5

Supplementary Data 6

Supplementary Data 7

Supplementary Data 8

Supplementary Data 9

Supplementary Data 10

Supplementary Data 11

Supplementary Data 12

Supplementary Data 13

## Data Availability

The Icelandic population WGS data has been deposited at the European Variant Archive under accession code PRJEB8636. The GWAS summary statistics are available at https://www.decode.com/summarydata. The data supporting the findings of this study are available within the article, its Supplementary Data files and upon request. The UK Biobank data can be obtained upon application (ukbiobank.ac.uk).
