# Supplementary material for: Sequence variants with large effects on cardiac electrophysiology and disease
Source: Nat Commun. 2019 Oct 22;10:4803. doi: 10.1038/s41467-019-12682-9 (PMC6805929; doi:10.1038/s41467-019-12682-9)
Supplement: Supplementary file 2 — Supplementary Information [file 41467_2019_12682_MOESM2_ESM.pdf]

Supplementary Information

Sequence variants with large effects on cardiac electrophysiology and disease

Norland et al.

Supplementary Figure 1

Pairwise Pearson correlations between the 123 QRS parameters tested. Amp.: amplitude; dur.: duration; QRS p-2-p: QRS peak-to-peak; VAT: ventricular activation time.

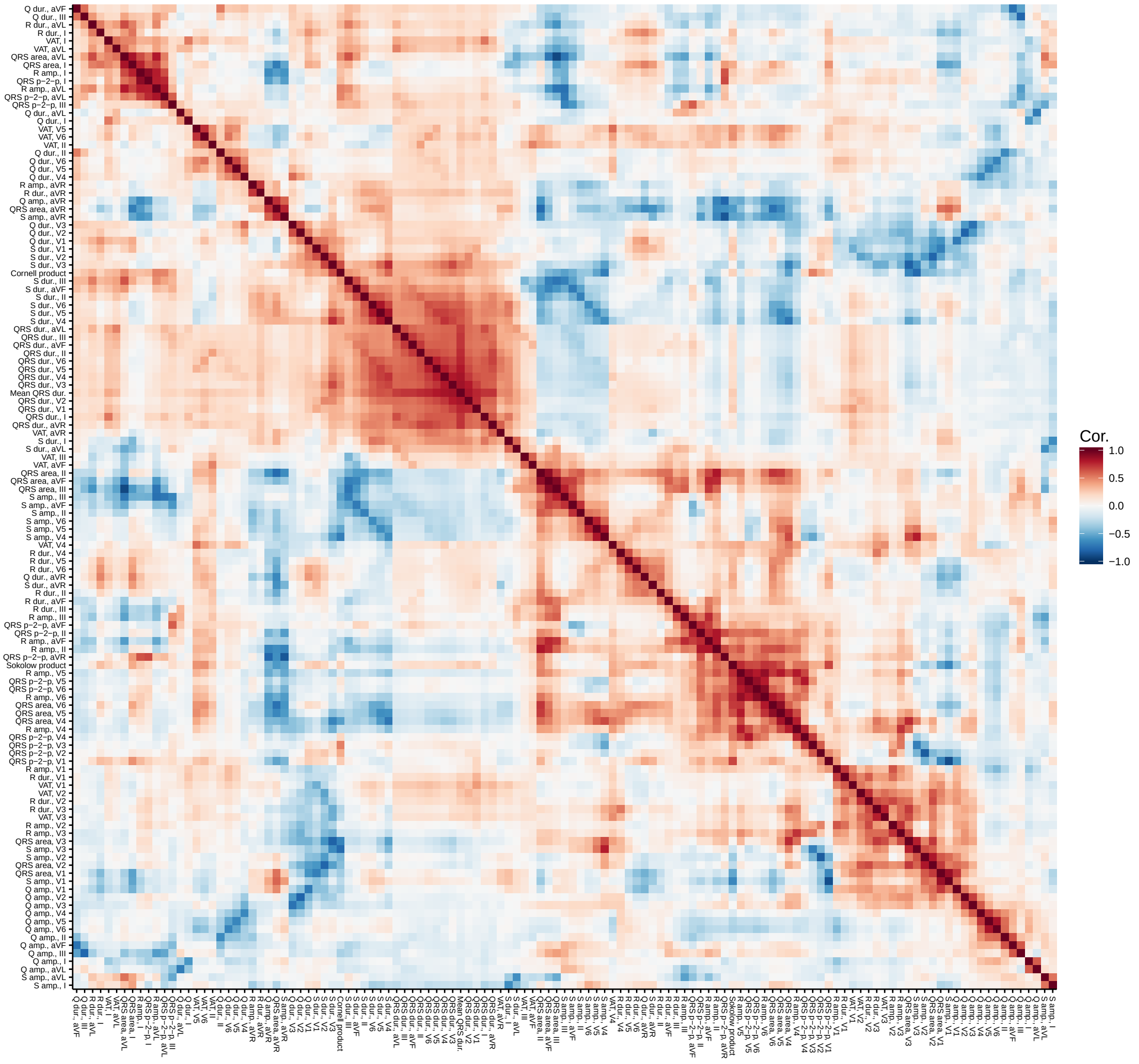

Supplementary Figure 2

Effects across the ECG (5% FDR, $P < 0.016$). The columns show the effects of QRS variants on ECG parameters, with non-significant parameters coloured white. We transformed the effects into a standard normal distribution using a rank-based transformation. For visualisation purposes, we flipped the direction of effects for some variants and omitted parameters that correlated with $(r^2 > 0.9)$ other parameters that had more significant associations. Amp.: amplitude; dur.: duration; QRS p-2-p: QRS peak-to-peak; VAT: ventricular activation time.

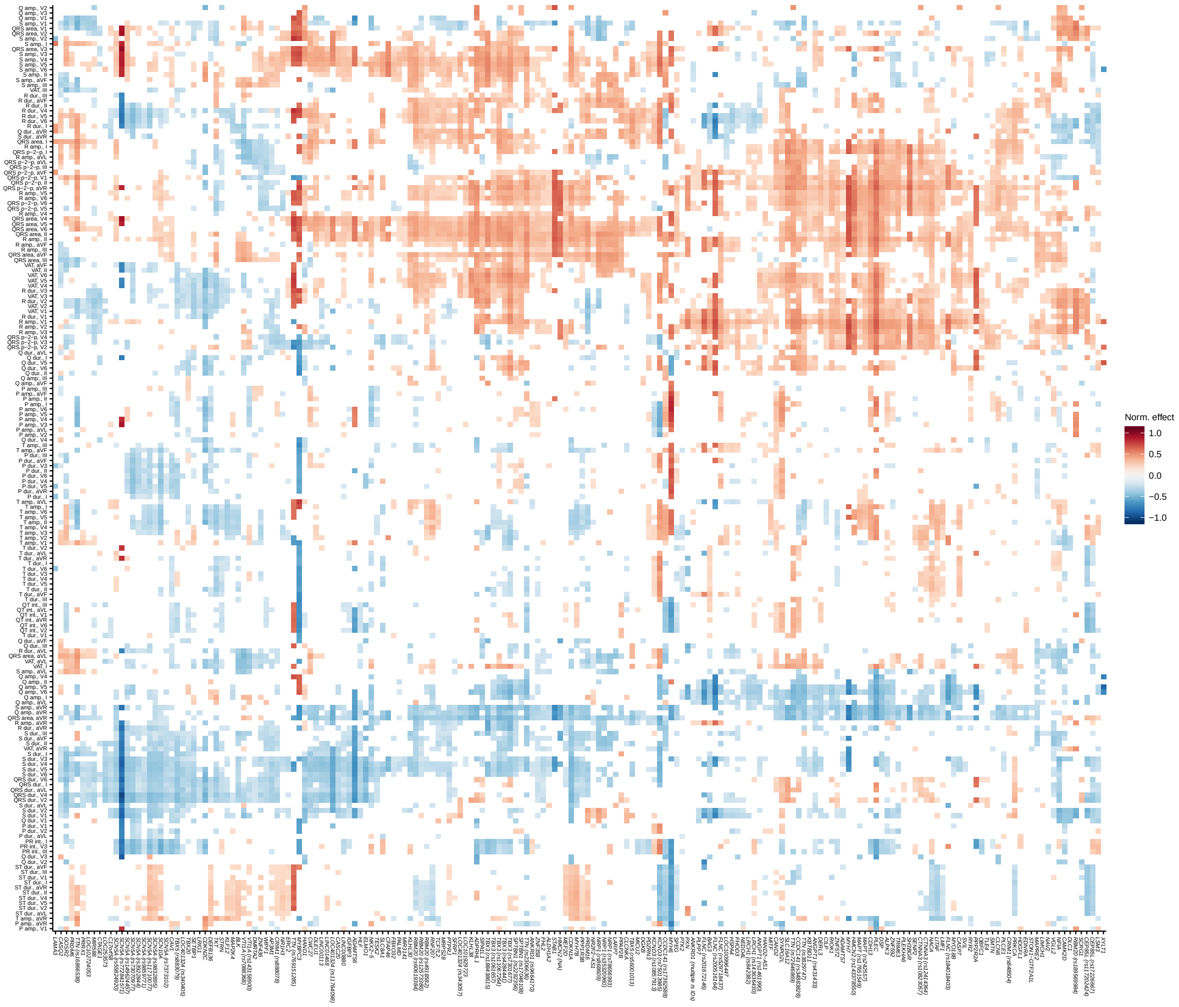

Supplementary Figure 3

Intersection plot of genome-wide significant $\left(P < 5 \times 10^{-8}\right)$ associations for all QRS measures. The left bar plot shows how many of the 190 QRS variants associate with each measure. The top bar plot shows the number of variants that associate with sets of measures denoted with (connected) black dots. Amp.: amplitude; dur.: duration; QRS p-2-p: QRS peak-to-peak; VAT: ventricular activation time.

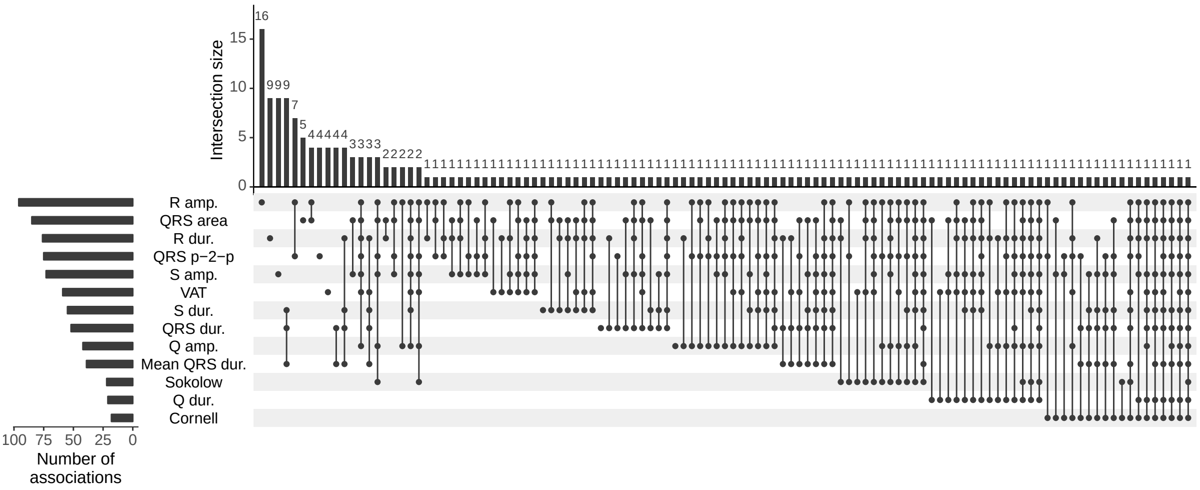

Supplementary Figure 4

Associations between variants that associate with AF and ECGs in sinus rhythm, excluding AF cases (5% FDR, $P < 0.018$). Amp.: amplitude; dur.: duration; int.: interval; QRS p-2-p: QRS peak-to-peak.

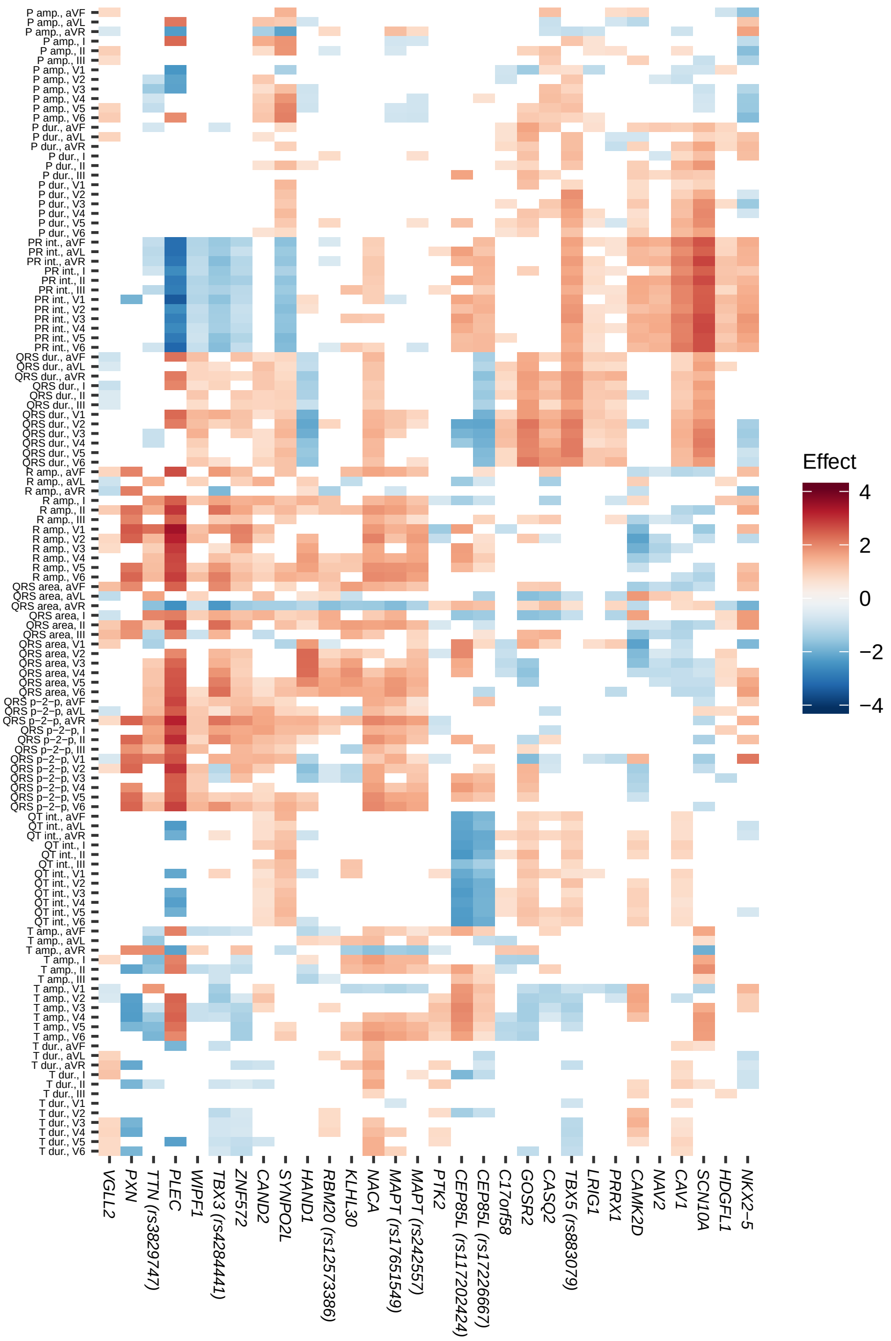

Supplementary Figure 5

Colocalization plot for expression of rs116904997 in *PXN*.

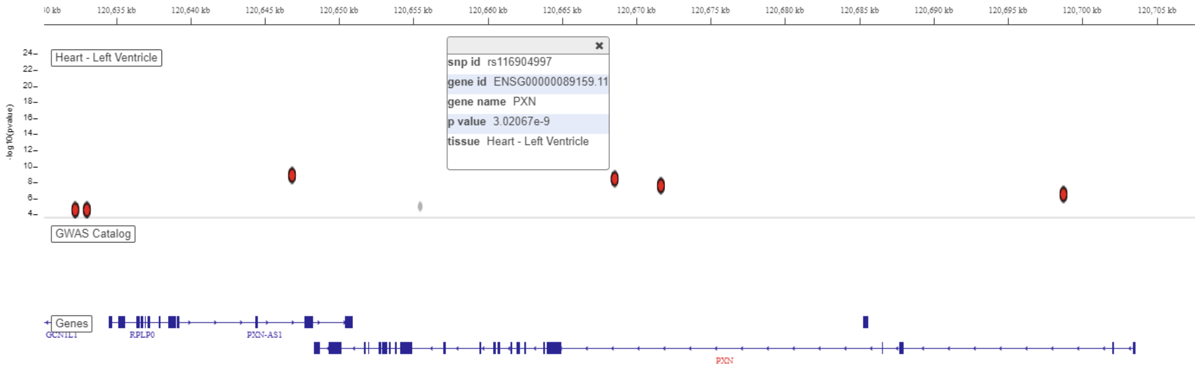

Supplementary Table 1. List of QRS complex parameters used in the study.

Phenotype	Sample size	Number of associations (P<5E-8)
QRS peak-to-peak, aVR	81186	47
R amplitude, V1	77889	47
QRS area, II	81016	43
QRS area, aVR	81108	40
R amplitude, II	81065	40
Mean QRS duration	81186	39
QRS duration, V3	81186	39
R amplitude, V2	80432	37
QRS duration, V4	81186	37
QRS duration, V2	81186	34
S duration, V4	78921	33
QRS duration, V5	81186	33
R duration, V5	81122	33
S duration, V5	74058	33
QRS area, aVF	80843	32
S amplitude, V5	74058	31
QRS duration, V1	81186	30
S amplitude, V4	78921	30
QRS area, V5	81063	30
QRS area, V4	80924	29
QRS area, V6	81110	29
Ventricular activation time, V6	81156	28
R amplitude, I	81161	27
S duration, V3	80246	27
S amplitude, aVR	49711	26
QRS duration, V6	81186	25
Ventricular activation time, V5	81122	24
Q amplitude, V6	55048	24
R duration, V6	81156	24
Q amplitude, aVR	56731	23
QRS peak-to-peak, I	81186	23
R duration, V4	81018	23
QRS area, I	81065	22
QRS peak-to-peak, II	81186	22
S duration, V2	79902	22
Sokolow-Lyon voltage criterion	76739	22
R amplitude, aVF	80517	21
R duration, V1	77889	21
R duration, V2	80432	21
R amplitude, V3	80849	20
QRS peak-to-peak, V6	81186	20
QRS peak-to-peak, V1	81186	19
S duration, V1	76834	19
Ventricular activation time, V4	81018	19
R amplitude, V6	81156	19
Cornell voltage criterion	79426	18
Ventricular activation time, I	81161	18
QRS area, III	80805	18
S amplitude, V3	80246	17
QRS duration, III	81186	16
QRS area, V3	80866	16
R duration, V3	80849	16
R amplitude, V4	81018	16
R amplitude, V5	81122	16

QRS area, V1	81100	15
QRS peak-to-peak, V3	81186	15
QRS area, aVL	80746	14
QRS duration, II	81186	14
QRS peak-to-peak, V2	81186	14
Ventricular activation time, V2	80432	14
S duration, V6	61135	14
S amplitude, V1	76834	13
QRS duration, aVF	81186	12
QRS duration, aVL	81186	12
R amplitude, aVL	80403	12
QRS duration, I	81186	12
R duration, I	81161	12
QRS area, V2	81000	12
Q amplitude, V5	44003	12
S amplitude, V6	61135	12
R duration, aVR	69796	11
Q amplitude, I	51543	11
S amplitude, III	59844	11
QRS peak-to-peak, aVF	81186	10
S amplitude, aVF	55661	10
QRS peak-to-peak, aVL	81186	10
QRS duration, aVR	81186	10
R duration, II	81065	10
S amplitude, V2	79902	10
Ventricular activation time, V3	80849	10
QRS peak-to-peak, V4	81186	10
R duration, aVF	80517	9
Ventricular activation time, aVR	69796	9
S amplitude, II	54431	9
Ventricular activation time, II	81065	9
R amplitude, III	79556	9
Ventricular activation time, V1	77889	9
Q duration, V6	55048	9
Q duration, aVR	56731	8
QRS peak-to-peak, III	81186	8
QRS peak-to-peak, V5	81186	8
Ventricular activation time, aVF	80517	7
R duration, aVL	80403	7
Ventricular activation time, aVL	80403	7
R amplitude, aVR	69796	7
S duration, I	48926	7
S duration, II	54431	7
S duration, aVR	49711	6
S amplitude, I	48926	6
S duration, aVF	55661	5
S duration, III	59844	5
Ventricular activation time, III	79556	4
Q duration, V5	44003	4
Q amplitude, aVL	52225	3
Q duration, I	51543	3
Q duration, V1	16702	3
S duration, aVL	50680	2
Q amplitude, II	45421	2
Q duration, II	45421	2
R duration, III	79556	2
S amplitude, aVL	50680	1
Q amplitude, III	45983	1
Q duration, III	45983	1

Q amplitude, V1	16702	1
Q amplitude, V4	25905	1
Q amplitude, aVF	43699	0
Q duration, aVF	43699	0
Q duration, aVL	52225	0
Q amplitude, V2	10807	0
Q duration, V2	10807	0
Q amplitude, V3	11762	0
Q duration, V3	11762	0
Q duration, V4	25905	0

Supplementary Table 2. Subject characteristics.

a) Subject characteristics and b) proportions of Icelanders that had ECG measurement taken from 1998 to 2015 and had cardiovascular disease by 2017.

a)

Variable	Value
Mean age (s.d.)	58.3 (20.1)
Number of males	41537
Number of females	39655

b)

Phenotype	%
Aortic valve stenosis	2.3
Atrial fibrillation	13
Coarctation of the aorta	0.061
Complete atrioventricular block	0.98
Congenital malformations of cardiac septa	0.62
Coronary artery disease	25
Dilated cardiomyopathy	0.46
Heart failure	11
Hypertension	36
Hypertrophic cardiomyopathy	0.36
Ischemic stroke	5
Mitral valve disease	0.93
Myocardial infarction	14
Pacemaker insertion	3.7
Patent ductus arteriosus	0.09
Perimyocarditis	1
Pre-excitation WPW	0.26
Sick sinus syndrome	3.1
Sudden cardiac death	2
Supraventricular tachycardia	2.24
Tetralogy of fallot	0.033
Ventricular tachycardia	1

Supplementary Table 3. GWAS Significance thresholds.

Adjusted thresholds for genome-wide significance from a weighted Bonferroni

Variant class	Number of variants	Significance threshold
High-impact variants	8464	2.60E-07
Moderate-impact variants	149983	5.10E-08
Low-impact variants	2283889	4.60E-09
Other variants in DNase I hypersensitivity sites	3913058	2.30E-09
Other	26108039	7.90E-10

Supplementary Table 4. Replication of reported QRS sequence variants.

Replication of reported QRS sequence variants at loci that we did not identify using our threshold for genome-wide significance.

Reported QRS parameter	Reference	deCODE QRS parameter	Variant	Chr.	Pos. (hg38)	Locus	Effect/other allele	EAF (%)	Annotation	Effect (s.d.)	*P*
12-lead sum	52 Genetic Loci Influencing Myocardial Mass	QRS area, II	rs12036340	1	162045950	*OLFML2B*	G/A	25.5	Intergenic	-0.03	3.40E-05
Cornell	52 Genetic Loci Influencing Myocardial Mass	QRS area, V4	rs10920184	1	201369768	*TNNT2*	T/C	44	Intronic	0.03	4.10E-06
Cornell	52 Genetic Loci Influencing Myocardial Mass	S duration, V4	rs6710065	2	26853689	*DPYSL5*	T/C	35.3	Intronic	-0.034	2.00E-07
QRS duration	52 Genetic Loci Influencing Myocardial Mass	S duration, V5	rs1344852	4	20182314	*SLIT2*	G/C	17.5	Intergenic	-0.033	7.70E-05
Sokolow-Lyon	52 Genetic Loci Influencing Myocardial Mass	QRS area, aVR	rs1015150	6	41691566	*TFEB*	T/C	44.7	Intronic	0.031	1.70E-06
QRS duration	52 Genetic Loci Influencing Myocardial Mass	QRS duration, V2	rs174577	11	61837342	*FADS2*	A/C	39	Intronic	-0.027	2.50E-05
Cornell	52 Genetic Loci Influencing Myocardial Mass	QRS peak-to-peak, V5	rs736825	12	54023792	*HOXC6*	G/C	39.7	Upstream	-0.023	2.60E-04
12-lead sum	52 Genetic Loci Influencing Myocardial Mass	QRS peak-to-peak, II	rs8038015	15	98720045	*IGF1R*	C/T	38.8	Intronic	-0.033	3.00E-07
12-lead sum	52 Genetic Loci Influencing Myocardial Mass	QRS peak-to-peak, aVR	rs7211246	17	30158744	*NSRP1*	G/A	40.9	Intronic	0.031	2.00E-06
12-lead sum	52 Genetic Loci Influencing Myocardial Mass	QRS peak-to-peak, aVR	rs7283707	21	15754675	*USP25*	A/G	13.2	Intronic	0.034	3.60E-04

Supplementary Table 5. List of echocardiographic traits used in the study.

Trait	Sample size
Aortic root diameter	19513
Ejection fraction	17109
Interventricular septum thickness	18775
Left ventricular end-diastolic diameter (LVEDD)	18487
Left ventricular end-systolic diameter	5704
Left ventricular posterior wall thickness	18626
Mitral Regurgitation	5291

Supplementary Table 6. List of cardiovascular diseases used in the study.

	deCODE		UK Biobank	
deCODE	deCODE cases	deCODE controls	UK Biobank cases	UK Biobank controls
Aortic valve stenosis	2457	349342	1735	405207
Atrial fibrillation	14710	373897	14792	393863
Coarctation of the aorta	119	355128	-	-
Complete atrioventricular block	1008	362214	585	406357
Congenital malformations of cardiac septa	1884	363696	571	407996
Coronary artery disease	38918	317957	28110	380457
Dilated cardiomyopathy	424	337689	653	406289
Heart failure	15237	384380	5406	403161
Hypertension	44290	306856	77566	331089
Hypertrophic cardiomyopathy	372	363446	131	403453
Ischemic stroke	5626	262087	3299	405268
Mitral valve disease	940	357390	324	403260
Myocardial infarction	24691	311354	14732	393835
Pacemaker insertion	3578	306162	2278	404664
Patent ductus arteriosus	628	357702	-	-
Perimyocarditis	971	343390	-	-
Pre-excitation WPW	275	347071	230	408425
Sick sinus syndrome	3568	346025	403	403181
Sudden cardiac death	3128	355146	926	407641
Supraventricular tachycardia	1461	348445	2349	406218
Tetralogy of fallot	60	352182	-	-
Ventricular tachycardia	945	351972	864	407703

Supplementary Table 7. FLNC associations.

			FLNC; **p.Ala2397Val**		***FLNC;*** **p.Phe1626Serfs***	
**Binary phenotype**	**Affected used**	**Controls used**	***P***	**OR**	***P***	**OR**
**MI**	23965	311807	1.30E-04	0.42	0.021	3.4
**MI < 76**	16763	319282	3.80E-06	0.21	0.011	4.1
**CAD all**	37782	318845	0.0049	0.60	1.50E-04	6.5
**CAD < 76**	25544	328262	7.00E-04	0.47	2.10E-05	7.8
**Dilated CMP**	412	335980	0.17	0	3.40E-10	85.4
**Quantitative trait**	**Sample size**		***P***	**Effect (s.d.)**	***P***	**Effect (s.d.)**
**Electrocardiogram R amplitude lead V1**	77889	-	5.00E-14	0.67	3.20E-04	-0.92
**MI age of onset**	23994	-	1.10E-04	0.78	0.041	-0.47
**CAD age of onset**	38970	-	0.051	0.28	0.028	-0.58

Supplementary Table 8. Parent-of-origin effects for the 190 QRS variants.

We only show variants with heterogeneity *P* <0.05.

								Maternal		Paternal		Mat. vs. Pat.	
**Top QRS parameter**	**Variant**	**Chr.**	**Pos. (hg38)**	**Effect/other allele**	**EAF (%)**	**Locus**	**Annotation**	**Effect (s.d.)**	***P***	**Effect (s.d.)**	***P***	I^2	***P* (heterogeneity)**
R duration, V4	rs11761424	7	14414730	A/G	35	*DGKB*	Intronic	0.02	0.034	0.07	6.50E-15	94.1	4.10E-05
R amplitude, V1	rs116904997	12	120230731	A/G	1.28	*PXN*	Intronic	0.07	0.062	0.28	7.30E-13	93	1.60E-04
QRS peak-to-peak, aVR	rs907683	2	219434819	G/T	44.2	*SPEG*	Upstream	0.06	9.40E-11	0.03	0.0038	85.6	0.0085
QRS duration, V4	rs3922844	3	38582762	T/C	32.2	*SCN5A*	Intronic	-0.03	7.00E-04	-0.06	1.30E-11	83.4	0.014
QRS duration, V4	rs9573330	13	73944073	A/G	31.4	*KLF12*	Splice region	-0.03	0.0081	-0.06	7.70E-10	82.9	0.016
Ventricular activation time, V3	rs500487	18	22505323	C/G	18	*CTAGE1*	Intergenic	-0.06	8.10E-09	-0.03	0.0072	80.4	0.024
R amplitude, V1	rs200516164	7	128856555	T/C	0.134	*FLNC*	Missense	0.48	6.80E-05	0.88	4.10E-11	80.2	0.025
R amplitude, I	rs55824920	3	38618461	T/C	31.4	*SCN5A*	Intronic	-0.03	0.0019	-0.06	7.70E-10	79	0.029
QRS area, aVL	rs12144451	1	115749108	C/T	43.1	*CASQ2*	Intronic	0.06	3.10E-13	0.04	1.30E-05	77.3	0.036
S amplitude, V4	rs61478147	5	154601099	A/G	34.8	*MIR3141*	Intergenic	-0.06	9.40E-10	-0.03	5.40E-04	75.3	0.044
QRS area, I	rs335196	5	123184837	A/G	43	*PRDM6*	Intronic	-0.03	1.10E-04	-0.06	2.10E-11	75.2	0.045
QRS peak-to-peak, V5	rs143378550	9	130307462	A/C	1.99	*HMCN2*	Stop gained	-0.15	1.00E-06	-0.24	9.80E-15	74.9	0.046
R amplitude, II	rs34967813	1	237678090	G/A	30.3	*RYR2*	Missense	-0.02	0.015	-0.05	2.90E-07	74.6	0.047
