# Supplementary material for: Sequence variants with large effects on cardiac electrophysiology and disease
Source: Nat Commun. 2019 Oct 22;10:4803. doi: 10.1038/s41467-019-12682-9 (PMC6805929; doi:10.1038/s41467-019-12682-9)
Supplement: Supplementary file 4 — Description of Additional Supplementary Files [file 41467_2019_12682_MOESM4_ESM.pdf]

Description of Additional Supplementary Files

File name: Supplementary Data 1

Description: Unreported associations between QRS parameters and sequence variants at 130 loci. We show the most significant association for variants that associate with multiple QRS parameters.

File name: Supplementary Data 2

Description: Associations, identified in conditional analyses, between QRS parameters and sequence variants.

File name: Supplementary Data 3

Description: Previously reported GWAS associations between sequence variants and the QRS complex.

File name: Supplementary Data 4

Description: Associations between the 190 QRS variants and automatic ECG diagnoses of four ventricular conduction disorders (5% FDR, $P \leq 0.003$). LAFB: Left anterior fascicular block (N_cases = 4034, N_controls = 336684). LBBB: Left bundle branch block (N_cases = 2628, N_controls = 283445). LPFB: Left posterior fascicular block (N_cases = 354, N_controls = 276364). RBBB: Right bundle branch block (N_cases= 2642, N_controls = 291972).

File name: Supplementary Data 5

Description: Associations between the 190 QRS variants and parameters across the entire ECG.

File name: Supplementary Data 6

Description: Replication of QRS variants in a dataset from a published GWAS, 52 Genetic Loci Influencing Myocardial Mass ($P<0.05$ and the same direction of effects).

File name: Supplementary Data 7

Description: Replication of QRS variants in UK Biobank data ($P<0.05$ and the same direction of effects).

File name: Supplementary Data 8

Description: Associations between the 190 QRS variants and echocardiographic traits ($P<0.05$).

File name: Supplementary Data 9

Description: Associations between the 190 QRS variants and cardiovascular diseases ($P<0.05$).

File name: Supplementary Data 10

Description: Tissue enrichment output from DEPICT (5% FDR).

File name: Supplementary Data 11

Description: Gene set enrichment output from DEPICT (1% FDR).

File name: Supplementary Data 12

Description: Gene prioritization output from DEPICT (5% FDR).

File name: Supplementary Data 13

Description: Associations between the QRS parameters and rare coding variants in genes with higher expression in the heart than other tissues (*P*<0.05/(number of coding variants tested)=2.4×10^{-5}).
