# Supplementary material for: Sequence variants with large effects on cardiac electrophysiology and disease
Source: Nat Commun. 2019 Oct 22;10:4803. doi: 10.1038/s41467-019-12682-9 (PMC6805929; doi:10.1038/s41467-019-12682-9)
Supplement: Supplementary file 3 — Peer Review File [file 41467_2019_12682_MOESM3_ESM.pdf]

Editorial Note: This manuscript has been previously reviewed at another journal that is not operating a transparent peer review scheme. This document only contains reviewer comments and rebuttal letters for versions considered at Nature Communications.

REVIEWERS' COMMENTS:

Reviewer #1 (Remarks to the Author):

The authors well responded to the comments of the reviewer. The current manuscript seems to be improved.We have no further comments.
